# Cancer anti-angiogenesis vaccines: Is the tumor vasculature antigenically unique?

**DOI:** 10.1186/s12967-015-0688-5

**Published:** 2015-10-29

**Authors:** Samuel C. Wagner, Thomas E. Ichim, Hong Ma, Julia Szymanski, Jesus A. Perez, Javier Lopez, Vladimir Bogin, Amit N. Patel, Francisco M. Marincola, Santosh Kesari

**Affiliations:** Batu Biologics Inc., Towne Center Drive, San Diego, CA 92121 USA; Pan Am Cancer Treatment Center, Tijuana, Mexico; Department of Surgery, University of Utah, Salt Lake City, UT USA; Sidra Medical and Research Center, Doha, Qatar; UCSD Moores Cancer Center, San Diego, CA USA

## Abstract

Angiogenesis is essential for the growth and metastasis of solid tumors. The tumor endothelium exists in a state of chronic activation and proliferation, fueled by the tumor milieu where angiogenic mediators are aberrantly over-expressed. Uncontrolled tumor growth, immune evasion, and therapeutic resistance are all driven by the dysregulated and constitutive angiogenesis occurring in the vasculature. Accordingly, great efforts have been dedicated toward identifying molecular signatures of this pathological angiogenesis in order to devise selective tumor endothelium targeting therapies while minimizing potential autoimmunity against physiologically normal endothelium. Vaccination with angiogenic antigens to generate cellular and/or humoral immunity against the tumor endothelium has proven to be a promising strategy for inhibiting or normalizing tumor angiogenesis and reducing cancer growth. Here we review tumor endothelium vaccines developed to date including active immunization strategies using specific tumor endothelium-associated antigens and whole endothelial cell-based vaccines designed to elicit immune responses against diverse target antigens. Among the novel therapeutic options, we describe a placenta-derived endothelial cell vaccine, ValloVax™, a polyvalent vaccine that is antigenically similar to proliferating tumor endothelium and is supported by pre-clinical studies to be safe and efficacious against several tumor types.

## Background

Angiogenesis, the outgrowth of new blood vessels from pre-existing capillaries and post-capillary venules, occurs during embryonic development, in the uterus during the menstrual cycle, in the process of wound healing, and in pathological conditions [1]. In healthy adults, endothelial cells can maintain a quiescent state for years, whereas they proliferate and migrate to form new vessels in response to inflammatory conditions and during tumor growth. Several studies have estimated that tumor associated endothelial cells proliferate 30–40 times faster relative to endothelial cells found in healthy vasculature [2–4]. Based on estimates that tumors fail to grow beyond 1–2 mm in the absence of new capillary growth, Dr. Judah Folkman put forth the central hypothesis that tumors release diffusible factors that stimulate endothelial cell proliferation in host capillary blood vessels [5]. Indeed, it has been estimated that eradication of one endothelial cell is capable of neutralizing of up to 100–300 tumor cells [6]. Since the immune system is in direct contact with the tumor vasculature, vaccination against tumor endothelium is theoretically very promising for breaching the immunological barriers created by the tumor microenvironment.

The goal in vaccination strategies is to raise immunity against antigens present in tumor endothelium while avoiding antigens that cross-react with healthy vasculature, thereby preventing deleterious autoimmune reactions. Since the landmark publication by Dr. Folkman, a catalog of molecular players involved in the process of tumor angiogenesis have been identified and characterized. However, clinical outcomes of traditional anti-angiogenic therapies such as monoclonal antibodies have improved patient survival rates only modestly [7]. Vaccination against endothelial cells is poised to overcome the existing problems of drug resistance and adverse side effects associated with other approaches. This report reviews vaccination strategies against the tumor endothelium that have been tested to date, including DNA, protein and peptide vaccines of tumor-endothelium-associated antigens, as well as polyvalent vaccines comprised of whole endothelial cells. Very encouraging data point toward the efficacy of vaccination in raising humoral and cell-mediated immunity against angiogenesis-associated antigens in cancer. In this discussion, we also highlight our novel approach wherein placenta-derived endothelial cell lysates (ValloVax™) are used as a source of antigen for vaccinating against proliferating tumor endothelial cells.

## How do angiogenic factors affect the tumor endothelium?

Endothelium is a dynamic and heterogeneous structure influenced by environmental factors such as shear stress, oxygen content of the blood, chemokines, cytokines, and changes in the content of the extracellular matrix [8]. Whereas resting endothelium serves to maintain blood fluidity, regulate blood flow, control vessel wall permeability, and quiesce circulating lymphocytes [9], environmental cues activate endothelial cells to proliferate, migrate, and form new branches (sprouting). In the tumor milieu, aberrantly elevated and chronic production of angiogenic factors leads to endothelial activation, vascular irregularities, and immune suppression, which are among the well-recognized hallmarks of cancer proliferation [10]. Whereas the structure of normal vascular endothelium is hierarchical and organized, the activated endothelium in cancer consists of dilated and tortuous blood vessels that are chaotically interconnected, leading to heterogeneous vessel density within the tumor, erratic blood flow, and focal regions of hypoxia [11, 12]. At the cellular level, tumor endothelial cells display a disorganized morphology, being loosely connected and exhibiting increased vascular permeability [13]. These conditions cause impaired oxygen and nutrient delivery, conditions which in turn trigger angiogenesis, thereby further promoting tumor endothelial cell activation and vascular growth to meet the metabolic demands of the tumor. In this manner, cancer cells and endothelial cells are involved in a positive feedback loop stimulating each other’s growth. At the same time, the vascular malformations in the tumor milieu serve as obstacles to effective penetration of anti-tumor lymphocytes and chemotherapy drugs into the tumor mass.

Although tumor endothelial cells are considered to be genetically stable as compared to tumor cells, angiogenic factors can fuel genetic aberrations of tumor endothelium. Indeed, resistance of tumor endothelial cells in diverse cancers has been described; for example, tumor endothelial cells in renal carcinoma are reportedly resistant to serum starvation [14], breast tumor endothelial cells are resistant to vincristine-induced apoptosis [15] and hepatocellular carcinoma endothelial cells are resistant to 5-fluorouracil and doxorubicin [16]. One study demonstrated aneuploid chromosomes and abnormal centrosomes in tumor endothelial cells [17]. Signaling in response to the dysregulated over-production of angiogenic factors, including vascular endothelial growth factor (VEGF) and fibroblast growth factor (FGF), in absence of endothelial stability promoting factors such as PDGF-BB was found to contribute to aneuploidy and centrosome duplication in tumor endothelial cells [18]. Moreover, tumor endothelial cells with excess centrosomes exhibit apoptosis resistance and formation of aberrant spindle projections, likely attributable to gain or loss of genes involved in proliferation, survival, and adhesion [18]. At the mechanistic level, VEGF secreted from tumors affects tumor endothelial cells by upregulating *MDR1*, a gene encoding a transmembrane glycoprotein P-gp that is known as a multidrug transporter that potentiates resistance to several anti-cancer agents [19]. For example, paclitaxel, which is used to treat several types of cancer together with anti-angiogenic drugs, is transported by P-gp. The VEGFR kinase inhibitor, Ki8751, and a phosphatidylinositol 3-kinase–Akt inhibitor, LY294002, effectively block tumor-induced MDR1 up-regulation, suggesting that VEGF in the tumor microenvironment is an underlying factor in acquired drug resistance [19]. Vaccination against angiogenesis-associated antigens therefore could serve as a valuable asset for improving the efficacy of other cancer therapeutics.

Heterogeneity among tumor endothelial cells, as choreographed by the over-activity of certain angiogenic pathways, also shields tumors by affecting their visibility to the immune system. Production of angiogenic molecules by tumors inhibits the expression of adhesion molecules involved in leukocyte interactions with blood vessel walls, including intercellular adhesion molecule-1 (ICAM-1), vascular cell adhesion molecule-1 (VCAM-1), E-selectin and CD34 [20]. These features of tumor endothelium prevent adhesion and extravasation of effector T cells into the tumor. Additionally, tumor endothelial cells have been described as anergic, marked by unresponsiveness to inflammatory cytokines that would normally induce adhesion molecule expression but instead allow the endothelium to escape immune surveillance [20, 21]. The anergic phenotype of tumor endothelial cells can be reversed by anti-angiogenesis therapy, which upregulates the expression of endothelial adhesion molecules in the tumor vasculature [22, 23]. The tumor endothelium is also prohibitive to entry of tumor-reactive T lymphocytes by suppression or direct killing of effector T cells via molecules such as Fas ligand (FasL) [24]. Notably, VEGF-A, interleukin 10 (IL-10) and prostaglandin E2 (PGE2) are involved in eliciting FasL expression in endothelial cells, as evidenced by observations that pharmacological inhibition of these molecules leads to a marked influx of tumor-rejecting CD8+ cells and tumor growth suppression in mice. Hence, anti-angiogenesis vaccination can break down the immunological firewall that the tumor endothelium imposes to protect the cancer.

## Approaches for vaccinating against tumor endothelium-associated antigens

Cancer vaccines consist of tumor-associated antigens delivered in a pro-inflammatory context to generate potent antitumor immune responses and overcome the cancer’s varied immunosuppressive mechanisms. Vaccination strategies directed against tumor-associated endothelium are designed to take advantage of both quantitative and qualitative differences between tumor endothelial cells and non-malignant endothelial cells. Ideal vaccine candidates include receptors/proteins that are upregulated in tumor endothelium, owing to its activated and proliferating state, but are sparsely expressed during physiological angiogenesis in healthy adult tissues. Notably, angiogenesis-associated molecules can be over-expressed on endothelial cells in an organ- or tumor-specific manner, and can sometimes be expressed on resting endothelial cells [25]. Thus, an attractive tumor endothelial vaccine should be capable of eliciting anti-angiogenesis and anti-cancer immunity against diverse tumor types, while avoiding autoimmune reactions against physiological angiogenesis, such that occur in the female reproductive tract and during wound healing, or against quiescent endothelium.

Whether immunity and/or autoimmunity ensue as a result of vaccination is dependent not only on the target tumor endothelial antigen but also on the vaccination protocol. Candidate vaccines have employed numerous delivery approaches, including proteins, peptides, dendritic cells pulsed with the antigen(s), naked DNA or recombinant DNA delivered by carriers, and mRNA vaccines, as well as whole cell vaccines or endothelial membrane components. To develop rationales for clinical vaccine design, pre-clinical studies have been conducted for many angiogenesis-associated molecules using various delivery systems. In some cases, differences in safety and efficacy between vaccines hinge on a plethora of secondary variables including the use of different delivery vectors, the choice of adjuvants and the varying routes of vaccine administration.

Two general approaches to anti-angiogenesis vaccination have yielded promising results for inducing specific and robust immunity against tumor endothelium and reducing tumor growth and metastasis: (1) vaccines expressing defined angiogenesis-associated antigens; and, (2) vaccines comprised of whole endothelial cells and/or mixtures of endothelial antigens. In the latter category are vaccines consisting of placenta-derived endothelial antigens, an approach currently being advanced by Batu Biologics, Inc. (ValloVax™ vaccine).

## Vaccines targeting defined angiogenesis-associated antigens

Evidence has shown the feasibility of targeting molecules that are expressed by angiogenic endothelium, and the data also suggest that anti-angiogenic vaccination can be applied synergistically with tumor immunotherapy and/or chemotherapy to invoke anti-tumor immunity. Theoretically, the approach of targeting certain defined angiogenesis-associated molecules on tumor endothelium has the risk to evoke only a transient decrease in cancer progression, owing to the fact that angiogenesis can proceed via compensatory pathways. Despite this caveat, success at achieving anti-angiogenic immunity has been demonstrated by vaccinating against the following antigens.

### VEGF-A and VEGFR

VEGF/VEGFR is perhaps the best-studied angiogenic pathway involved in the growth and survival of tumor endothelium. Clinical therapies addressing tumor angiogenesis have therefore largely focused on inhibiting VEGF and its cognate receptor. VEGF-A exists in several pro-angiogenic variants that are secreted by both tumor cells and endothelial cells and activate VEGFR1 and VEGFR2. Numerous VEGF-expressing vectors have been administered for vaccinating against VEGF. A *Xenopus* VEGF DNA vaccine that was protective and therapeutic in several tumor models in mice, an effect that was mediated by anti-tumor activity of CD4+ lymphocytes [26]. A phase I clinical study has investigated CIGB-247, a vaccine comprising a human VEGF variant molecule in combination with a bacterial adjuvant, in 30 patients with advanced solid tumors [27]. After eight consecutive weeks of subcutaneous immunization and re-vaccination on week 12, this vaccine was safe at three dose levels and also demonstrated immunogenicity in three sequential analyses of patients’ blood samples. Despite the critical importance of VEGF in hematopoiesis [28–30], no abnormalities in leukocytes or megakaryocytes were found, and additionally, serum biochemistry parameters were not altered in patients received the VEGF vaccination. Based on this clinical report, VEGF is a highly promising vaccine target as a cancer treatment strategy. The implications of this body of research are that immunity can be induced to self-antigens, without triggering a fulminant autoimmune response.

VEGFR2 (also FLK-1 and KDR), which is highly expressed on proliferating endothelial cells of the tumor vasculature, has also been the focus of numerous pre-clinical studies. Animal models using VEGFR2 DNA, protein, and peptide vaccines have demonstrated their ability to elicit potent humoral and cellular immunity, suppression of angiogenesis, tumor necrosis and/or suppression of metastastatic progression [31–38]. Interestingly, none of the aforementioned studies have reported hematopoietic or other abnormalities.

Since VEGFR2 is also expressed at lower levels in normal vascular endothelium, vaccination could theoretically cause side effects or an autoimmune response. Neithammer et al. reported that in murine models a DNA vaccine against VEGFR2 suppressed angiogenesis in the tumor vasculature as verified by in vitro inhibition of endothelial cell proliferation, deposition of antibodies in tumor vasculature, a reduction of microvessel density and antitumor activity in vivo without impairment of fertility, neuromuscular performance or hematopoiesis; however, a slight delay in wound healing in immunized mice was noted [39]. In another report, there was no delay in wound healing observed in response to a dendritic cell-based VEGFR2 vaccine although reduced litter sizes and fetal loss were found in vaccinated animals [40]. However, in other studies where VEGFR2 protein vaccines were found to generate potent immunity against tumor endothelium, no effects on wound healing, reproduction or other organ toxicities were observed [35, 41].

Immunogenic epitopes of VEGFR2 have been identified and peptide vaccines have been optimized for binding to MHC class I molecules in order to elicit activity of cytotoxic T lymphocytes (CTL) specific for tumor endothelium. In a clinical study of pancreatic cancer, administration of immunogenic VEGFR2-169 peptide vaccine in combination with gemcitabine was well tolerated with no severe adverse events and peptide-specific CTL were induced [42]. Other than injection site reactions, other peptide vaccines tested for targeting VEGFR were also deemed to have manageable toxicities [43–45].

### bFGF and FGF-R

Basic fibroblast growth factor (bFGF or FGF-2) and its receptor (FGFR-1, CD331) have been targeted in pre-clinical mouse models to evaluate their anti-angiogenic and anti-tumor effects. FGFR-1 is highly expressed on angiogenic endothelium as well as on endothelial progenitor cells [46, 47]. In one such study, vaccination with xenogeneic FGFR-1 plasmid DNA inhibited tumor endothelial cell proliferation and produced anti-cancer immunity in three murine tumor models [48]. Studies utilizing recombinant protein vaccines targeting FGFR-1 demonstrated significantly decreased tumor volume compared to controls, as well as decreased microvessel density in tumors without any observable overt toxicity [49]. Peptide bFGF vaccines combined with adjuvants also induced antigen-specific antibody and cell-mediated responses [50, 51]. Importantly, in a study where a liposome-based peptide bFGF vaccine was administered in murine cancer models, immunity against tumor endothelium was elicited while physiological angiogenesis was unperturbed, as evidenced by normal wound healing times and no impairments in the reproductive ability of vaccinated animals or in the viability or health of the offspring [52].

### αvβ3

The expression of several cell adhesion molecules, most notable integrin alpha(v)beta(3), has been associated with tumor angiogenesis [53].

Integrin αvβ3 plays a role in several physiological processes including angiogenesis, pathological neovascularization, and tumor metastasis [53]. An αvβ3 ligand vaccine induced a humoral response associated with significant antitumor activity without recourse of adjuvant therapy. The immune response was driven by antibody-dependent cellular toxicity and complement-directed cytotoxicity to the tumor-associated endothelial cells [54]. The effectiveness of utilizing antibodies blocking αvβ3 as a method of inhibiting angiogenesis, has led to the clinical development of monoclonal anti-αvβ3 antibodies for the treatment of advanced solid tumors. MEDI-522, a second generation humanized anti-αvβ3, successfully completed a Phase I study in patients with a variety of metastatic solid tumors, and a maximum tolerated dose was not identified by traditional dose-limiting toxicities [55].

### Angiomotin

Another target of anti-angiogenic vaccine strategies is the angiostain binding protein angiomotin, a membrane-associated pro-angiogenic protein present on endothelial cells in angiogenic tissues [56]. Holmgren et al. used DNA vaccination with a xenogeneic (human) angiomotin DNA construct to increase immunogenicity of the vaccine, and injected the DNA intramuscularly followed by electroporation [57]. This vaccine protected mice from tumor challenge and acted synergistically with a vaccine targeting the Her-2 oncogene in a transgenic breast cancer model system. No evidence for autoimmunity in normal vasculature of the retinas was noted at 16 or 70 weeks post-treatment. In another report, an angiomotin DNA vaccine was shown to alter blood vessel architecture to increase permeability and thus improve the efficacy of chemotherapy in an animal model [58].

### Endoglin (CD105)

This transmembrane glycoprotein is primarily expressed in proliferating endothelial cells and is upregulated by hypoxia; therefore, endoglin is strongly expressed in tumor endothelial cells [59]. The functional role for endoglin in hematopoiesis is also exemplified by CD105 knockout studies where embryonic lethality occurred as a result of impaired angiogenesis in the yolk sac and heart defects. CD105 expression on tumor vessels is a prognostic factor correlated with poor overall and disease-free survival, tumor recurrence, and metastasis of various cancers [60, 61]. CD105 vaccination approaches employing bacterial surface display of protein [62] and orally administered DNA vaccines [63] effectively targeted the vasculature and inhibited tumor growth in the absence of observable effects on healthy tissues.

### Survivin

This is an intracellular tumor-associated antigen that is upregulated in cancers and tumor endothelial cells but is not expressed in healthy differentiated tissues. Survivin is a member of the inhibitor of apoptosis protein family, and serves to inhibit programmed cell death, promote cellular proliferation and enhance angiogenesis [64]. Survivin vaccines elicit apoptosis of tumor cells and its vasculature with variable efficacy; while intradermal electroporation was found to be very effective in animal models, naked DNA administration is less effective in the prophylactic or therapeutic settings [65, 66].

### Robo4

Robo4 is a member of the Roundabout family proteins and is a transmembrane cell adhesion molecule that is specifically expressed in endothelial cells and hematopoietic stem cells and progenitor cells [67]. Robo4 guides the formation of the vasculature by controlling endothelial cell migration and coordinating blood vessel sprouting [68]. Tonic Robo4 signaling in healthy tissues maintains vascular integrity by inhibiting VEGF-induced endothelial cell migration and vascular permeability [69]. Robo4 is pathologically over-expressed in the tumor endothelium [70], it promotes atypical vascular patterning that reduces blood-tumor barrier permeability, thereby likely impeding the access of chemotherapy drugs to tumors [71]. In one study, a protein vaccine against this target was developed, comprised of the extracellular domain of mouse Robo4, fused to the Fc domain of human immunoglobulin within an adjuvant. Vaccinated mice had a strong antibody response to Robo4, impaired fibrovascular invasion in vitro, and reduced growth of implanted Lewis lung carcinoma [72]. Further studies will be needed to determine how breaking tolerance to Robo4 affects physiological angiogenesis, where it appears to counteract angiogenic signaling [73].

### Tie-2/angiopoietin receptor

Tie-2 is an endothelium-specific receptor tyrosine kinase that binds to angiopoietin and is upregulated on diseased endothelium in diverse conditions including cancer, atherosclerosis, and macular degeneration. A protein vaccine based on chicken Tie-2 reduced growth of hepatomas and melanomas in mice, whereas a murine Tie-2 vaccine was ineffective, indicating the need for further enhancement of this protein’s immunogenicity [74]. The chicken Tie-2 vaccine exhibited apoptotic and anti-angiogenic effects on endothelial cells. The ligand for Tie-2, angiopoietin-2 is also over-expressed during neoangiogenesis in tumors and acts synergistically with VEGF in promoting tumor vascularization [75].

### EGFR

**Fig. 1:**
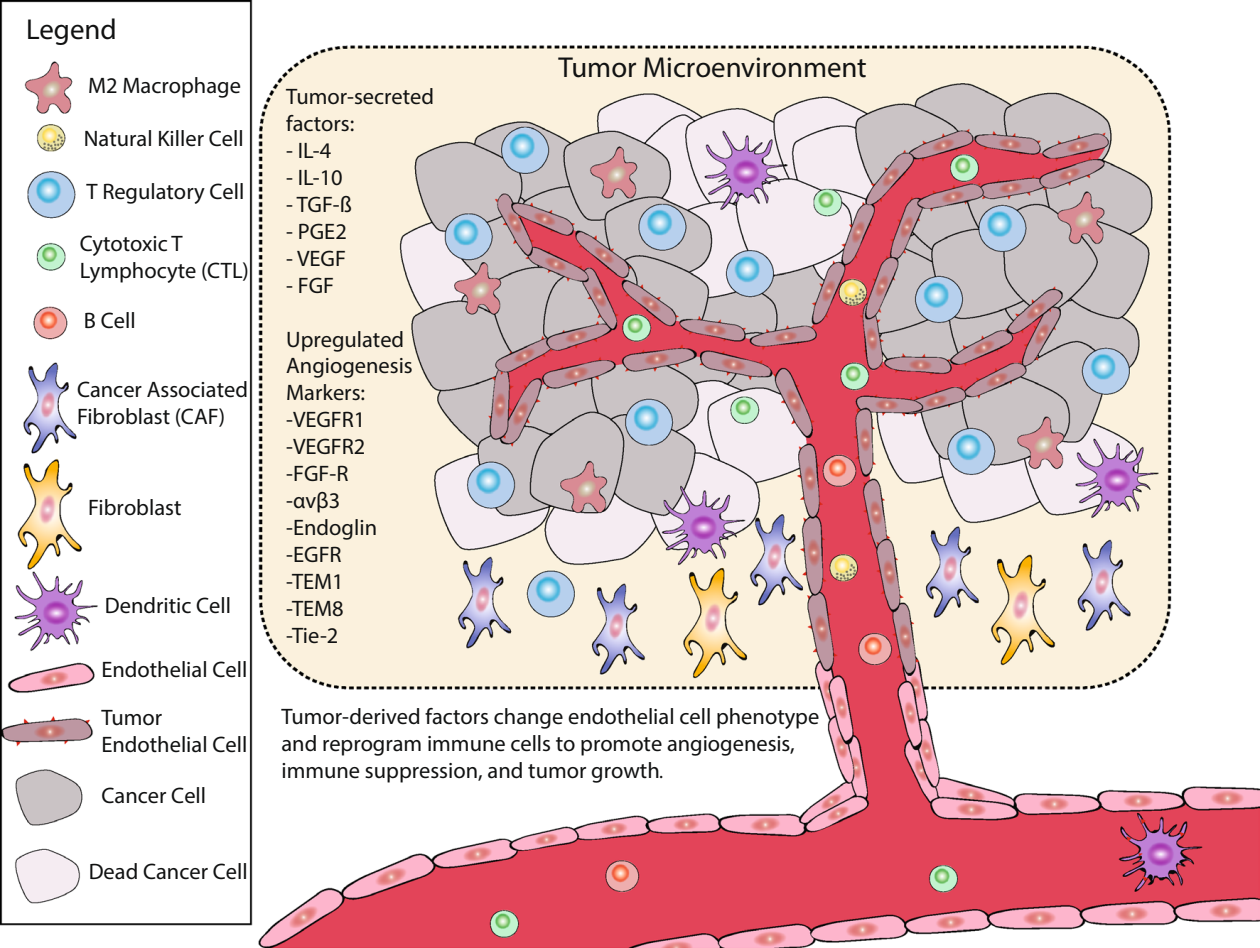
Unique structure of tumor vasculature. The tumor vasculature is identified by several irregular characteristics: they are leaky, fragile with many holes and tears, and have chaotic blood flow. These structural differences in the tumor endothelial cells reflect a fundamental change in endothelial cell phenotype when in the presence of tumor secreted factors such as: VEGF, TGF-B, IL-4, IL-10, FGF, and many other cytokines/growth factors. This change in phenotype can be characterized by the upregulated expression of several related angiogenesis markers that have relatively low expression on healthy endothelium in vivo

This receptor can be activated by diverse ligands; namely, EGF, transforming growth factor-α (TGF-α), amphiregulin, betacellulin, heparin-binding EGF, or epiregulin that are locally secreted by cancer cells and can act in an autocrine fashion. EGFR signaling regulates the secretion of several different angiogenic growth factors by tumor cells including VEGF [76]. Co-expression of EGFR ligands and EGFR is associated with malignant tumor phenotypes and poor prognosis in cancer patients [77–79]. Monoclonal antibodies and vaccines targeting this pathway have been implemented clinically, including the monoclonal antibodies cetuximab, panitumumab and nimotuzumab; the small tyrosine kinase molecules erlotinib and gefitinib; the EGF-based cancer vaccine CIMAvax^®^; and a EGFR-based HER-1 cancer vaccine. CIMAvax-EGF is a therapeutic cancer vaccine of human recombinant epidermal growth factor (EGF) conjugated to a carrier protein from *Neisseria meningitides.* The vaccine induces antibodies against EGF and confers a survival advantage in patients with non-small-cell lung cancer [80]. Studies of post-operative wound healing revealed that there are no deleterious effects on physiological angiogenesis associated with targeting the EGF pathway [81] (Fig. 1).

### HP59

This polypeptide is expressed as a marker for neonatal lung vasculature and adult pathological angiogenesis. HP59 protein has been identified in the vasculature of many types of tumors including lung, colon, ovary, and breast cancers of different stages, but not in the corresponding normal tissues; therefore, this protein could be an ideal target antigen for vaccinating against tumor endothelium [82]. Vaccination of mice with immunogenic HP59 peptides attenuated the growth of Lewis lung tumors and inhibited pathological angiogenesis, as evidenced by an absence of HP59-expressing blood vessels. HP59 possesses a 41-amino acid sequence at the NH_2_ terminal that has no homology with any known protein; therefore, this antigen could be used for raising specific immunity against the pathological vasculature in human cancer [82].

### PDGFRβ

This tyrosine kinase receptor is activated by members of the platelet-derived growth factor family, and is broadly involved in regulating cellular proliferation and differentiation in physiological and pathological contexts. PDGF-B, the ligand, produced by tumor cells binds to PDGFR on stromal and perivascular cells to promote tumor growth and angiogenesis, as well as eliciting other pathways of tumor nourishment [83, 84]. PDGFRβ supports angiogenesis in two distinct ways: (1) by activating pericytes, which support endothelial cell proliferation and elaboration of the tumor vasculature; and (2) by upregulating expression of the proangiogeneic factor FGF-2 [84].

Anti-PDGF drugs have been widely used for treating various cancers; however, their mechanisms of action at a molecular level are not strictly anti-angiogeneic. Paradoxically, PDGF ligand inhibition can increase tumor angiogenesis, which supports increased tumor growth but, in doing so, normalizes blood vessel walls to allow for more efficient delivery of chemotherapy drugs [85, 86]. On this basis, inhibiting the PDGF pathway can serve as an effective adjunct to cytotoxic drugs and yield a net gain in eradicating tumors [85].

To assess the effects of vaccinating against PDGFRβ, a DNA vaccine delivered via *Salmonella typhimurium* was given to mice orally in murine colon, breast and lung carcinoma model systems [87]. Significantly, in a therapeutic setting where tumors were implanted 20 days before vaccination, the size of tumors in the PDGFRβ-immunized group was one-fourth the size of the control group. Angiogenic markers within tumors were reduced and robust cytotoxic T cell responses against PDGFR-β were detectable, indicating that angiogenesis was effectively targeted.

### TEM1

Tumor endothelial marker 1, also known as endosialin or CD248, is a type 1 membrane protein involved in developmental, physiological and pathological angiogenesis. TEM1 is overexpressed in the vasculature of carcinomas, brain tumors and sarcomas [88–91] and is present on endothelial cells, pericytes, and fibroblasts [92, 93]. A DNA vaccine expressing full-length mouse *Tem1* cDNA fused to tetanus toxoid adjuvant inhibited tumor growth and progression without affecting reproduction or wound healing [94]. Splenocytes from TEM1 vaccinated mice secreted IFN-γ and lysed TEM1-expressing endothelial cells in vitro. These data support the use of TEM1 protein vaccine in conjunction with an adjuvant to break tolerance to tumor endothelium for the treatment of solid tumors.

### TEM8

Designing a tumor endothelial vaccine based on TEM8 is a very enticing possibility since this molecule is selectively upregulated on tumor endothelial cells and, unlike the majority of other putative vaccine targets, TEM8 is undetectable in endothelium during physiological angiogenesis [95]. The first TEM8 DNA vaccine was a syngeneic vaccine that was tested in a rat model of breast cancer and murine melanoma [96]. Although the vaccine had no activity as a single agent, TEM8 DNA significantly enhanced anti-tumor immunity when administered with a rat HER2 DNA vaccine for breast cancer, and also in combination with a tyrosine-related protein-1 DNA vaccine in melanoma. Ruan et al. developed a xenogeneic TEM8 DNA vaccine carried by xenogeneic *Salmonella typhimurium* that was capable of generating TEM8-specific CD8+ cytotoxic T cells and protected mice from lethal tumor challenge [97]. Another vaccination approach consisted of dendritic cells transduced with recombinant adenovirus encoding TEM8, which also effectively protected mice from lethal challenges against hepatocellular carcinoma [98]. Lastly, a DNA vaccine encoding syngeneic TEM8 and murine beta-defensin 2, which activates dendritic cells to stimulate potent immunity, was used to inhibit tumor growth in a murine colon cancer model [99]. This vaccine was also highly effective, causing the collapse of tumor vessels by evoking an antigen specific CD8+ T cell response. Further studies are warranted to determine whether TEM8-based vaccines can be safe and efficacious for clinical use.

In summary, several therapeutic approaches have been explored targeting single antigen expressed on the tumor endothelium, demonstrating the feasibility of targeting the tumor vasculature by vaccine therapy without inducing systemic autoimmunity to quiescent endothelium in vivo. Signals of efficacy have been observed in preclinical studies around these targets, and several tumor endothelium-targeting vaccines have made it into the clinical setting for the treatment of cancer. As such, in an effort to reduce the development of treatment resistance and to potentiate the strength of an immune response and resultant survival benefit, the authors would like to explore the concept of a polyvalent vaccine targeting several angiogenesis associated antigens.

## Endothelial cell vaccines

Although great strides have been made in advancing anti-angiogenesis tumor vaccines against specific targets, it is clear that the presence of many interrelated and compensatory pathways, as well as genetic instability of tumor endothelium could theoretically overcome targeted inhibition in a clinical setting. Accordingly, polyvalent vaccination approaches are being developed using whole endothelial cells or isolated proteins from endothelial cell membranes. Another advantage of such polyvalent vaccines expressing numerous angiogenic antigens is to allow antigen-presenting cells to process and present immunodominant epitopes for generating anti-angiogenic immunity.

### Preclinical studies of endothelial cell vaccines

Human umbilical vein endothelial cells (HUVEC) have been the standard for cell-based models of tumor angiogenesis, having the ability to proliferate extensively and expressing a number of pro-angiogenic molecules that mimic tumor neovasculature. Specifically, the aforementioned antigens such as VEGFR2, αvβ3 and endoglin, which are common biomarkers associated with tumor angiogenesis, are expressed in the primary culture of HUVECs [100]. In a report by Wei et al. [100], paraformaldehyde fixed xenogeneic whole HUVEC used as a vaccine markedly inhibited tumor growth in prophylactic and therapeutic murine cancer models. The anti-angiogenic effect of this vaccine depended on CD4+ T cells eliciting endothelial-specific antibody responses. Using a syngeneic vaccine consisting of fixed hepatic sinusoidal endothelial cells, Okaji et al. also demonstrated potent preventative and therapeutic anti-tumor immunity in a lung metastasis model of murine colon cancer [101]. Both antibody and cytotoxic T cell responses against endothelial cells were detected in response to this vaccine. Similar studies performed in animal models have reported potent angiogenesis inhibition and tumor targeting when using syngeneic endothelial cell vaccines [102, 103], xenogeneic endothelial cells [104], and xenogeneic endothelial proteins [105]. Although the experimental design differences between these studies preclude any definitive conclusions concerning the optimal vaccine candidate, these data collectively provide a compelling proof of concept that tolerance to tumor endothelium can be broken using whole endothelial cells as vaccines.

### Clinical progress with endothelial cell vaccines

HUVECs have been used in pilot studies to test the anti-angiogenic effects of vaccination in patients with malignant brain tumors and metastatic colorectal cancer [106, 107]. Vaccinations were performed using 5 × 10(7) HUVECs given intradermally on a weekly basis for the first month and the every 2 weeks from the second month onward. In a published report where a total of 230 vaccinations were administered to 6 patients with recurrent malignant brain tumors and 3 patients with metastatic colorectal cancer, MRI results showed partial or complete responses lasting for a minimum of 9 months in 3 of the patients with brain tumors [106]. Moreover, antibodies directed against HUVEC antigens were detected in eight out of nine patients and HUVEC-specific CTLs were detected in 6 of 7 tested patients. No adverse events were reported with the exception of skin reactions at the vaccine injection site.

In a related study, 17 patients with recurrent glioblastoma were treated with the HUVEC vaccine using the same protocol of intradermal delivery [107]. These patients had been previously treated with surgery and chemoradiotherapy, and were also undergoing salvage treatments including concomitant and adjuvant chemotherapy during the course of the study. The results showed that HUVEC vaccine therapy is feasible for recurrent glioblastoma based on significant prolongations of the tumor doubling times and reduced tumor growth rates at 3- and 6-month follow ups. Despite the fact that 352 vaccinations were performed, no adverse events were observed with the exception of skin reactions at the injection sites. For comparative purposes, the investigators point out that bevacizumab, a humanized monoclonal antibody to VEGF, is associated with grade 3 adverse events in 46.4 % of patients when used alone and in 65.8 % of patients when used in combination with chemotherapy [108], demonstrating that a HUVEC vaccine appears to be much safer than conventional anti-angiogenesis drug therapies [107]. The authors conclude that, for invasive and large tumors, HUVEC vaccination is feasible for use in combination with other treatment modalities and similar trials should be conducted for other types of cancer.

## Placental endothelial cell vaccines: advancing ValloVax™ for cancer treatment

Among the vaccine candidates for targeting tumor endothelium, the placenta is a source of significant numbers of proliferating and angiogenic cells owing to its immunological status at the maternal-fetal interface. In the placenta, trophoblast cells invade into the maternal uterine wall where they remodel the spiral arteries and supply angiogenic factors for de novo blood vessel formation and expansion of the pre-existing vascular network [109]. These processes must meet the high demands for blood supply and nutrient exchange of the growing fetus, analogous to the elaborate vascular networks required for tumor growth.

The idea of using placenta as a source of antigens for vaccination against tumors originated in the 1970s from Dr. Valentin Govallo who noted the immunological similarities between pregnancy and cancer and demonstrated that immunity to placental trophoblast cells conferred radiological tumor reductions in lung cancer patients (reviewed in [110]). Indeed, a substantial body of literature validates numerous parallels in proliferation, angiogenesis, invasion, and immune suppression between cancer and pregnancy that is attributable to shared characteristics of fetal-derived trophoblast cells of the placenta and tumor cells [111]. Common angiogenesis-associated molecules that have been identified as highly expressed in tumor cells and in placenta include VEGF family members [112], FGF/FGFR, survivin [113], calreticulin [114], angiomotin [115], ROBO4 [116], and PDGFB/PDGFR [117]. Placenta is also a practical source for purification of endothelial cells for therapeutic purposes with yields reported from 2 to 10 billion primary endothelial cells per placenta [118–120]. In contrast, the theoretical yield of total cells from umbilical cord (including mesenchymal and endothelial cells) is 500 million cells with reported yields of less than half of this amount following isolation and processing [121]. Placental endothelial cells are thus a feasible alternative to HUVECs as vaccines for targeting tumor vasculature.

Batu Biologics is currently advancing ValloVax™, a vaccine consisting of placenta-derived endothelial cells pretreated with interferon gamma to enhance immunogenicity. Pre-clinical studies revealed that ValloVax™ potently inhibits tumor growth in 3 histologically distinct animal models and also suppresses pulmonary metastasis subsequent to intravenous tumor administration [122]. This new approach awaits clinical validation and may serve as a novel anti-angiogenesis vaccine that can be deployed against a variety of tumor types.

## Conclusions

Blocking tumor-induced angiogenesis has become a focal point for the development of new cancer therapeutic drugs. Vaccines represent a promising approach for overcoming tolerance to the angiogenic factors that support cancer growth and metastasis. Antigens that are highly expressed on proliferating tumor endothelium, while comparatively downregulated or absent on quiescent endothelium in healthy tissues and during normal physiological angiogenesis are ideal candidates for vaccination strategies targeting the tumor vasculature. Two major types of vaccines are being developed; namely, vaccines against defined angiogenesis-associated antigens, and whole endothelial cell vaccines. The data available thus far support the feasibility of these approaches for generating humoral and cell-mediated immunity against the tumor vasculature and the safety of endothelial cell-based vaccines.
